# Attosecond photoionization delays in the vicinity of molecular Feshbach resonances

**DOI:** 10.1126/sciadv.ade3855

**Published:** 2023-04-12

**Authors:** Vicent J. Borràs, Jesús González-Vázquez, Luca Argenti, Fernando Martín

**Affiliations:** ^1^ Departamento de Química, Módulo 13, Universidad Autónoma de Madrid, 28049 Madrid, Spain.; ^2^ Institute for Advanced Research in Chemical Sciences (IAdChem), Universidad Autónoma de Madrid, 28049 Madrid, Spain.; ^3^ Department of Physics and CREOL, University of Central Florida, Orlando, FL 32186, USA.; ^4^ Instituto Madrileño de Estudios Avanzados en Nanociencia (IMDEA Nano), Cantoblanco, 28049 Madrid, Spain.; ^5^ Condensed Matter Physics Center (IFIMAC), Universidad Autónoma de Madrid, 28049 Madrid, Spain.

## Abstract

Temporal delays extracted from photoionization phases are currently determined with attosecond resolution by using interferometric methods. Such methods require special care when photoionization occurs near Feshbach resonances due to the interference between direct ionization and autoionization. Although theory can accurately handle these interferences in atoms, in molecules, it has to face an additional, so far insurmountable problem: Autoionization is slow, and nuclei move substantially while it happens, i.e., electronic and nuclear motions are coupled. Here, we present a theoretical framework to account for this effect and apply it to evaluate time-resolved and vibrationally resolved photoelectron spectra and photoionization phases of N_2_ irradiated by a combination of an extreme ultraviolet (XUV) attosecond pulse train and an infrared pulse. We show that Feshbach resonances lead to unusual non–Franck-Condon vibrational progressions and to ionization phases that strongly vary with photoelectron energy irrespective of the vibrational state of the remaining molecular cation.

## Teaser

Coupled electronic and nuclear motions in molecular Feshbach resonances reflect in attosecond photoionization delays.

## INTRODUCTION

Since its inception at the dawn of the 21st century, one of the goals of attosecond science has been to access the natural time scale of electronic motion, in the hope of being able to image in real time and eventually manipulate the early stages of electron-driven processes, as, e.g., charge transfer and electron transport (*1*–*3*), photo-induced radiation damage (*4*–*6*), or photo-induced electron transport reactions (*7*, *8*), to name a few. Among other things, attosecond techniques have opened the way to determine the time an electron takes to escape from atoms after absorption of a photon with large enough energy to overcome the binding potential (*9*–*14*), the so-called attosecond photoionization delay (*15*, *16*). Recent experimental and theoretical work (*17*–*25*) suggests that photoionization delays may also be obtained for molecules and solids by extending the tools already developed for atoms, but how this should be done in practice or how the results of the measurements should be interpreted are questions that are still under close scrutiny.

Photoionization delays are mostly determined from interferometric methods, such as the reconstruction of attosecond beatings by interference of two-photon transitions (RABBIT) (*26*, *27*). In this method, a harmonic comb, associated with an XUV attosecond pulse train (APT), is combined with an ultrashort femtosecond infrared (IR) pulse to induce two-photon two-path interferences from which relative phases, hence relative photoionization delays, can be obtained by varying the time delay between the APT and the IR. This technique has been successfully used to obtain photoionization delays in atoms in a wide range of photoelectron energies (*9*, *12*, *14*, *28*, *29*) and to accurately determine the abrupt phase variations occurring in the vicinity of resonances lying in the electronic continuum of atoms (*14*, *28*, *29*). In many cases, the analysis and interpretation of the experimental results required the help and guidance from accurate theoretical calculations (*14*, *28*, *29*), which, in the case of atoms, are available to describe not only one-photon ionization but also the two-photon ionization processes at play. The latter is not a trivial matter, since the second photon is absorbed when the electron is already in the continuum, so that continuum-continuum transitions must be evaluated. In contrast, applications to molecules are much scarcer, mainly due to the additional complication introduced by the nuclear degrees of freedom. By using RABBIT, photoionization phases and/or delays have already been measured for simple molecules such as CO, N_2_, N_2_O, H_2_O, and CF_4_ (*17*, *23*–*25*, *30*). They have also been measured for the ethyl iodide molecule using attosecond streaking techniques (*21*). These experiments considered regions of the photoelectron spectra where resonances are absent or barely populated [CO, H_2_O, CF_4_, and ethyl iodide (*17*, *21*, *30*)] or where broad, short-lived shape resonances show up [N_2_O, N_2_, and CF_4_ (*23*–*25*, *30*)]. In these cases, the experimental results were successfully interpreted by either assuming that the nuclei remain fixed during the ionization process or by making use of the Born-Oppenheimer approximation. Both approaches are justified by the fact that, in the photon energy ranges that were considered, the nuclei move much more slowly than the escaping electron. However, molecules, as atoms, are plagued with Feshbach resonances, especially at low photoelectron energies. Many of these resonances have long autoionization lifetimes, so that the nuclei have enough time to move substantially from their initial positions before the electron is emitted (*31*–*33*). As a consequence, the fixed-nuclei and Born-Oppenheimer approximations do not work anymore, and one has to account for the coupling between electronic and nuclear motions.

The presence of Feshbach resonances has been shown to have marked consequences in the determination of photoionization phases in the context of RABBIT and streaking experiments performed in the N_2_ and H_2_ molecules (*22*, *34*, *35*), where not only the energy (and eventually the ejection direction) of the electron must be measured (as in atoms), but also the vibrational state of the remaining molecular cation (nondissociative ionization) or the kinetic energy of the ionic fragments (dissociative ionization), as well as the molecular orientation with respect to the light polarization direction, must be determined. For a correct interpretation of these interferometric measurements, theoretical calculations that account for both the electronic and nuclear motions, the coupling between them, and electron correlation responsible for the autoionizing decay and the bound-continuum and continuum-continuum transitions induced by the XUV and IR pulses are mandatory. So far, this has only been achieved for the H_2_ molecule (*22*, *35*).

This is likely the reason why RABBIT experiments performed in molecules have ignored the region of Feshbach resonances. The only exception is the pioneering experimental work of Haessler *et al.* (*34*), reported in 2009, where the observation of unusual vibrational progressions in the RABBIT spectrum of N_2_ was tentatively attributed to the presence of a Feshbach resonance. This prediction was supported by simulations based on a simple one-dimensional square-well potential model. Thus, to further advance in our understanding of photoionization delays in molecules containing many electrons, advanced theories capable of describing molecular autoionization while the nuclei move (as those available for H_2_) are needed. Here, we present an accurate theoretical approach that fulfills these requirements, and we apply it to study RABBIT in the vicinity of the N_2_ Feshbach resonances by using a combination of pulses as in the experiment of Haessler *et al.* (*34*).

We show that Feshbach resonances are responsible for unusual non–Franck-Condon vibrational progressions and abrupt phase variations, modulated by the nuclear motion, in the RABBIT spectra of N_2_. The presence of a forest of long-lived Feshbach resonances favors the production of molecular cations in higher vibrational states than in regions where Feshbach resonances are absent and leads to an apparent jump in the photoionization delays of up to 600 as. This is the result of the superposition of different autoionization channels in the low-energy region of the spectrum. On the other hand, wider Feshbach resonances, as the lowest ones in the so-called Hopfield series (*36*), leave a clear signature in the measured photoionization delays. We show that information about such resonances can be better retrieved from angularly resolved RABBIT spectra for a fixed molecular orientation (i.e., in the molecular frame). Current experimental efforts are pursuing this goal by combining RABBIT setups with multicoincidence detection methods (*22*, *23*, *25*, *35*) or by aligning the molecule with an auxiliary laser [see, e.g., (*37*)].

## RESULTS AND DISCUSSION

**Fig. 1.**
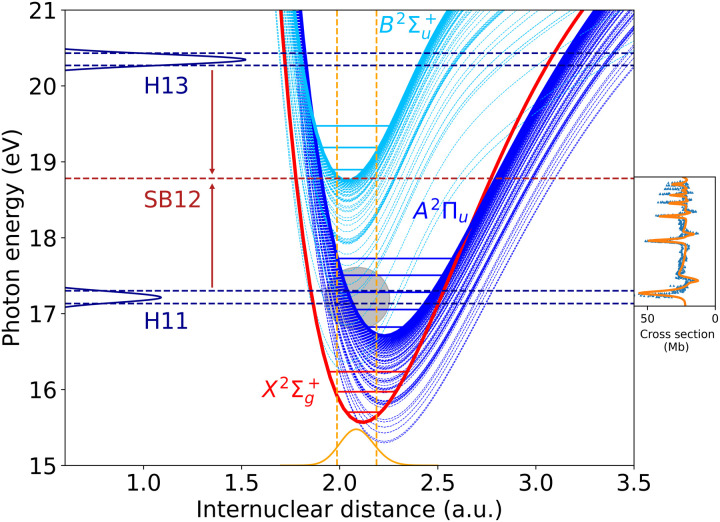
The electronic continuum of N_2_. Potential energy curves of the $X^{2}{\text{Σ}}_{g}^{+}$ (thick red line), *A*^2^Π*_u_* (thick blue line), and $B^{2}{\text{Σ}}_{u}^{+}$ (thick pale blue line) states of $N_{2}^{+}$ and the corresponding Feshbach resonances of N_2_ converging to the former three states (thin lines). The horizontal lines contained within the potential energy curves of the $X^{2}{\text{Σ}}_{g}^{+}$, *A*^2^Π*_u_*, and $B^{2}{\text{Σ}}_{u}^{+}$ states indicate the position of the lowest vibrational levels in each state. The Gaussian-like curve (orange) at the bottom of the figure represents the *v* = 0 vibrational state in the ground electronic state of N_2_. The vertical dashed lines in orange represent the boundaries of the Franck-Condon region. The horizontal blue peaks on the left-hand side of the figure show the harmonics H11 and H13 associated with the APT used in the calculations, and the dashed horizontal lines in blue show the the energy region where one expects that Feshbach resonances are populated by these harmonics. The semitransparent gray shaded circle highlights the region where resonances are expected to be populated by absorption of an H11 photon. The two red arrows indicate the emission and absorption of an IR photon, from H11 and H13, respectively. The dashed red line indicates the position of the sideband SB12 that results from the two interfering processes mentioned above. The small panel on the right-hand side of the figure shows the one-photon ionization spectra, adapted from figure 3 of (*44*), as measured at synchrotron facilities (*38*–*41*) and calculated by Klinker *et al.* (*44*).

Figure 1 shows the calculated potential energy curves for the three lowest ionization channels, $X^{2}{\text{Σ}}_{g}^{+}$, *A*^2^Π*_u_*, and $B^{2}{\text{Σ}}_{u}^{+}$, and the Feshbach resonances converging to the latter two channels (notice that some of these resonances become true bound states when they cross the lowest ionization threshold $X^{2}{\text{Σ}}_{g}^{+}$). These resonances have been observed in synchrotron radiation (*38*–*41*) and attosecond (*42*) experiments. In these experiments, resonances converging to the $B^{2}{\text{Σ}}_{u}^{+}$ threshold, the so-called Hopfield series (*36*), exhibit the usual Fano-like profiles resulting from the interference between direct one-photon ionization and autoionization (*43*). The corresponding one-photon ionization spectrum associated with these resonances (*38*–*42*, *44*) is displayed on the right of the figure. An accurate theoretical description of the Hopfield series has only been recently achieved (*44*, *45*) by means of the XCHEM method (*46*, *47*), which correctly accounts for electron correlation in the molecular continuum responsible for the autoionizing decay. Figure 1 also shows the positions of the H11 and H13 harmonic bands produced in the experiment of Haessler *et al.* (*34*) and the two interfering paths leading to the SB12 sideband lying in between. In the first path, an H11 XUV photon and an IR photon are absorbed, while in the second path, an H13 XUV photon is absorbed, and IR photon is emitted. Both paths lead to electrons with the same energy and are therefore indistinguishable. Within the Franck-Condon region, H11 can populate both the higher Feshbach resonances converging to the *A*^2^Π*_u_* threshold, and the lowest Feshbach resonances of the Hopfield series lying just above that threshold (i.e., converging to the $B^{2}{\text{Σ}}_{u}^{+}$ threshold). All these resonances will leave their imprint in the SB12 sideband and consequently will affect the interference between the two paths, hence the photoionization phases.

**Fig. 2.**
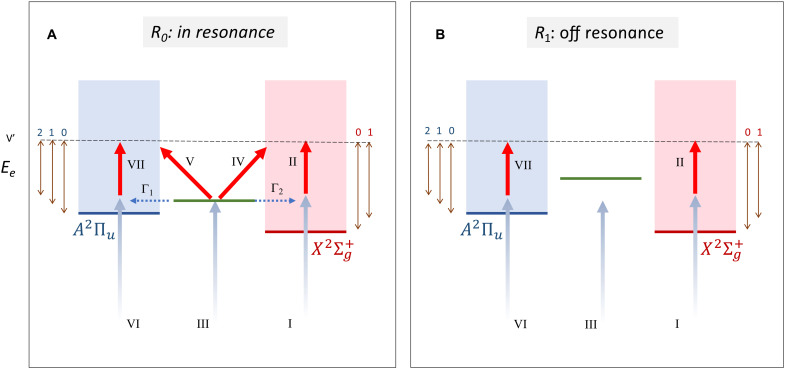
Sketch of the ionization paths involving a Hopfield resonance of N_2_. (**A**) On-resonance scenario at *R* = *R*_0_. (**B**) Off-resonance scenario at *R* = *R*_1_. Horizontal green line: energy position of the resonance. Blue shaded area: ionization continuum associated with the *A*^2^Π*_u_* state of $N_{2}^{+}$. Red shaded area: ionization continuum associated with the $X^{2}{\text{Σ}}_{g}^{+}$ state of $N_{2}^{+}$. Blue arrows represent absorption of a single XUV photon from the ground state, and red arrows represent absorption of an IR photon. Blue dotted arrows represent autoionization of the resonance into the *A*^2^Π*_u_* and $X^{2}{\text{Σ}}_{g}^{+}$ channels through the Γ_1_ and Γ_2_ paths, respectively. Vertical double arrows indicate the accessible photoelectron energies when the cation is left in the different vibrational states with quantum numbers 0, 1, 2, etc., and for the total energy indicated by the black horizontal dashed line. Roman numbers indicate steps of the ionization paths in which a photon is either absorbed or emitted.

A sketch of the different ionization paths that are accessible when an H11 photon populates a Hopfield resonance is shown in Fig. 2. When such a resonance (green horizontal line) is resonantly populated at a given internuclear distance *R*_0_, in addition to paths I + II and VI + VII contributing to SB12 after absorption of an IR photon in the nonresonant continuum (one path per ionization channel), four additional paths are also possible: III + Γ_2_ + II, III + Γ_1_ + VII, II + IV, and II + V. In the first two, the IR photon is absorbed by the electron that results from autoionization of the resonance, while in the second two, the IR photon is absorbed from the resonance before autoionization, i.e., it is absorbed by the neutral molecule. All these six paths plus the two paths corresponding to absorption of an H13 photon and stimulated emission of an IR photon lead to ionization states with the same total energy and, therefore, interfere with each other. The total energy available in the system after ionization can be shared between the emitted photoelectron and the remaining molecular cation in different ways, leading to vibrational progressions of peaks in each ionization channel instead of to a single peak in the photoelectron spectrum. As the nuclei move and the internuclear distance changes to *R*_1_, it may be that the Hopfield state is no longer resonantly excited because of a variation of its energy position with respect to that of the ground state, so that only paths I + II and VI + VII and the corresponding ones associated with the higher H13 harmonic survive. In a dynamical situation where the nuclei move during the interaction with the APT and the IR pulse, both scenarios coexist.

In this work, we have extended the XCHEM methodology to solve the time-dependent Schrödinger equation (TDSE) in the presence of the APT and the IR pulse by accounting for the nuclear motion in a full quantum mechanical way (see Methods), so that all of the above processes are correctly described. Adiabatic potential energy curves and electronic wave functions for bound, continuum, and Feshbach resonances were evaluated in a dense grid of internuclear distances and then transformed into the corresponding diabatic counterparts to account for nonadiabatic effects in a numerically stable way. All bound-bound, bound-continuum, and continuum-continuum involving these diabatic states were explicitly evaluated. We have used a duration of 25 fs [full width at half maximum (FWHM)] for the IR pulse and 300 as (FWHM) for each pulse contained in the APT. The corresponding intensities are 5 × 10^11^ W/cm^2^ and 3.5 × 10^12^ W/cm^2^, respectively. More details are given in Methods.

**Fig. 3.**
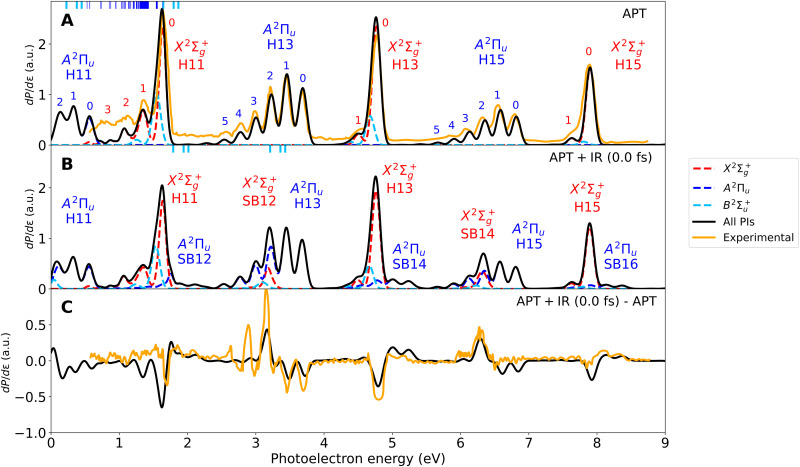
N_2_ photoelectron spectra. Using (**A**) the APT only and (**B**) the APT and the IR with zero delay between them. (**C**) Difference between (A) and (B). Black curves: total spectrum. Dashed colored curves: contribution of the different ionization channels ($X^{2}{\text{Σ}}_{g}^{+}$, red; *A*^2^Π*_u_*, blue; $B^{2}{\text{Σ}}_{u}^{+}$, pale blue). Orange curves: experimental results from (*34*). The small number on top of the different peaks indicates the vibrational state in which the molecular cation is left in each ionization channel. The small vertical lines on top of (A) and (B) indicate the position of the Feshbach resonances lying below (blue) and above (pale blue) the *A*^2^Π*_u_* threshold. Theoretical spectra have been convoluted with a Gaussian of 80 meV FWHM to account for the experimental resolution of (*34*). a.u., atomic units.

Figure 3A shows the calculated and experimental photoelectron spectra resulting from the APT only. As can be seen, the overall agreement between theory and experiment is very good. The figure also shows the individual contributions of the different ionization channels. The harmonic bands associated with the *A*^2^Π*_u_* channel are well separated from those associated with the $X^{2}{\text{Σ}}_{g}^{+}$ and $B^{2}{\text{Σ}}_{u}^{+}$ channels, which overlap in the same spectral region, especially at the lowest photoelectron energies. The H13 and H15 bands associated with the *A*^2^Π*_u_* channel, hereafter called H13(*A*^2^Π*_u_*) and H15(*A*^2^Π*_u_*) for short, exhibit a well-defined and extended vibrational progression, from *v*′ = 0 to *v*′ = 6, with relative peak intensities that closely follow the Franck-Condon overlaps between the initial vibrational state and the final vibrational states in the *A*^2^Π*_u_* state of the cation. The vibrational progressions for the harmonic bands associated with the $X^{2}{\text{Σ}}_{g}^{+}$ and $B^{2}{\text{Σ}}_{u}^{+}$ channels are much narrower, with a very dominant peak for *v*′ = 0 and a tiny one for *v*′ = 1, as dictated by the corresponding Franck-Condon overlaps. In contrast, as pointed out in (*34*), the H11$\left(X^{2}{\text{Σ}}_{g}^{+}\right)$ band exhibits what seems to be a wider vibrational progression, with a much more pronounced *v*′ = 1 peak and the appearance of at least two additional peaks where one would expect to find peaks associated with the *v*′ = 2 and *v*′ = 3 vibrational states of the $X^{2}{\text{Σ}}_{g}^{+}$ electronic state. This is the energy region where Feshbach resonances are populated by the H11 harmonic (see Fig. 1). To facilitate the identification of the Feshbach resonances that contribute in this energy region, Fig. 3A includes short vertical lines in the top horizontal axis, indicating the positions where these resonances are expected to appear according to our calculations. As can be seen, a forest of Feshbach resonances converging to the *A*^2^Π*_u_* ionization threshold is potentially responsible for the unusually high intensities of the *v*′ = 1, *v*′ = 2, and *v*′ = 3 peaks. The spectrum of Fig. 3A also shows that the H11(*A*^2^Π*_u_*) band does not follow the Franck-Condon behavior observed in the H13(*A*^2^Π*_u_*) and H15(*A*^2^Π*_u_*) bands [experiments of Haessler *et al.* (*34*) did not have access to this energy region]. Of course, the vibrational progression is truncated at *v*′ = 2, i.e., at almost zero photoelectron energy, because there is not enough energy available for the escaping electron when the cation is left in higher vibrational states. However, independently of this, one can see a substantial increase in intensity and a notorious widening of the *v*′ = 2 peak compared to the corresponding peak in the H13(*A*^2^Π*_u_*) and H15(*A*^2^Π*_u_*) bands. Both effects are the consequence of populating the lowest three Feshbach resonances of the Hopfield series, which lie just above the *A*^2^Π*_u_* ionization threshold. As can be seen in the photoionization spectrum shown in Fig. 1, these Feshbach resonances are nearly degenerate with the *v*′ = 2 state of the *A*^2^Π*_u_* channel, which explains why this is the vibrational peak that is more seriously affected by their decay into the *A*^2^Π*_u_* state. Furthermore, the first and the third resonances in the Hopfield series, *ns*σ*_g_* and *nd*σ*_g_*, have the largest autoionization widths [∼0.06 and ∼0.1 eV, respectively, corresponding to lifetimes of ∼10 and ∼7 fs, see (*45*)], so that their decay occurs, while the nuclei are moving. These resonances overlap with each other and with the second resonance of the Hopfield series, *nd*π*_g_*, leading to a structure with an apparent width of ∼0.2 eV (see the photoionization spectrum on the right panel of Fig. 1), hence the widening of the *v*′ = 2 peak in the H11(*A*^2^Π*_u_*) band. The lowest Hopfield resonances can also decay into the $X^{2}{\text{Σ}}_{g}^{+}$ state, producing faster electrons that contribute to the signal around the *v*′ = 0 peak in the H11$\left(X^{2}{\text{Σ}}_{g}^{+}\right)$ band. This is indicated in the figure with the corresponding short vertical line on the top horizontal axis. However, this is barely seen in the spectrum of Fig. 3A due to the large intensity of the nonresonant process leading to *v*′ = 0 in the $X^{2}{\text{Σ}}_{g}^{+}$ state and to the fact that autoionization decay into the $X^{2}{\text{Σ}}_{g}^{+}$ state is less likely than into the *A*^2^Π*_u_* state (*44*), which is closer in energy. In contrast, as we will see below, this same resonant decays into *v*′ = 0 of the $X^{2}{\text{Σ}}_{g}^{+}$ state and has an enormous effect on the photoionization phases.

Figure 3B shows the photoelectron spectrum obtained by combining the APT and the IR pulse at zero delay. As discussed above, the presence of the IR field leads to the appearance of sidebands in between the harmonic bands. For the chosen pulses, the sidebands associated with a given ionization channel completely overlap with the harmonic bands of the other channels. For example, SB12$\left(X^{2}{\text{Σ}}_{g}^{+}\right)$ and SB12$\left(B^{2}{\text{Σ}}_{u}^{+}\right)$ overlap with H13(*A*^2^Π*_u_*), and SB12(*A*^2^Π*_u_*) overlaps with H11$\left(X^{2}{\text{Σ}}_{g}^{+}\right)$. Spectral overlap between harmonic bands and/or sidebands associated with different ionization channels is the common rule in all molecules due to the vibrational broadening of these bands and the relatively small energy differences between electronic states of the molecular cation. Both effects are obviously absent in atomic systems. For photoelectron energies larger than 4 eV, the observed vibrational progressions resulting from the overlap between harmonic bands and sidebands associated with different channels are nearly identical to those reported at higher energies (*24*). At these higher energies, the relative intensities of the different peaks can be perfectly explained in the framework of the Born-Oppenheimer approximation, as shown in (*24*). However, this is not the case below 4 eV. Once again, this is due to the presence of the Feshbach resonances, whose signature not only shows up in the lower harmonic bands but also can be transferred to the neighboring sidebands. To better identify the contribution of the sidebands, experimentalists usually subtract the photoelectron spectra obtained with the APT only from that obtained with both the APT and the IR. This is shown in Fig. 3C, where the calculated spectrum is compared with the measured one in (*34*). As can be seen, the general agreement is good, although, in this case, the experimental spectrum is more noisy and does not display the smallest features so clearly.

We note that, in contrast with the photoionization spectrum of atoms or the spectrum shown in the right-hand side of Fig. 1 for N_2_, the presence of autoionizing resonances in this region does not lead to peaks with Fano profiles. There are several reasons for this. The first one is the convolution of the calculated spectra to account for the limited energy resolution in the experiment, which is 80 meV. The largest resonance width, 98 meV [see (*44*)], corresponds to the lowest state of the Hopfield series. All of the other resonances have much smaller widths, so that, after convolution, their contribution spreads out over the 80 meV interval. The second reason is that Feshbach resonances do not necessarily lead to standard Fano profiles in vibrationally resolved photoelectron spectra due to the coupling with nuclear motion [see, e.g., (*32*, *33*)]. This coupling can spread out the signature of the resonances over an interval of photoelectron energies, which can be wider than that associated with the actual autoionization width, due to the decay of the resonances over a range of internuclear distances. The third reason is the spectral width of the harmonics. In the experiment of Haessler *et al.* (*34*), this spectral width is comparable to or larger than the autoionization width of most resonances, so that Fano-like profiles, as those arising from ionization with perfectly monochromatic light, can be notably distorted. In contrast, the spectrum shown in the right inset in Fig. 1 was measured by using monochromatic synchrotron radiation, and the energy resolution was much higher. We also note that the inset shows the ionization cross section as a function of photon energy, not photoelectron energy (as it is the case for the RABBIT spectrum shown in Fig. 3B). At a given photon energy, the photoionization spectrum of Fig. 1 contains contributions from all final vibrational levels, so that the effect of the nuclear degrees of freedom is less visible.

To make the above points clearer, fig. S1 shows the photoelectron spectra plus the population remaining in the Feshbach resonances when the TDSE is integrated up to 50 fs, and no convolution is performed. In this short time interval, the accessible resonances converging to the *A*^2^Π*_u_* threshold (blue curves in Fig. 1) appear as narrow spikes because they have not had enough time to decay by autoionization (their lifetimes are much longer than the integration time), while those lying just above the *A*^2^Π*_u_* threshold (Hopfield resonances, pale blue curves in Fig. 1) have completely decayed. As can be seen, between 0.5 and 1.5 eV photoelectron energy, many sharp peaks are observed. They correspond to the many resonances converging to the *A*^2^Π*_u_* threshold that can be accessed by the 11th harmonic in the Franck-Condon region and can therefore decay to the $X^{2}{\text{Σ}}_{g}^{+}$ continuum (see Fig. 1). After they autoionize, their contribution spreads over wider energy intervals, which, in combination with the convolution performed to account for the limited energy resolution of the experiment, prevents one from resolving them individually. However, in any case, they are collectively responsible for the unusual vibrational progressions observed at those low energies. Thus, at variance with the interpretation given in (*34*), the non–Franck-Condon behavior in the H11$\left(X^{2}{\text{Σ}}_{g}^{+}\right)$ band is not due to a single Feshbach resonance but to a collection of them. Only two resonances lying above the *A*^2^Π*_u_* threshold (the lowest members of the Hopfield series) are energetically accessible and leave their signature in both the $X^{2}{\text{Σ}}_{g}^{+}$ (at ∼1.7 eV) and *A*^2^Π*_u_* (at ∼0.1 eV) continua. In the presence of the IR, some of these resonances (Hopfield and non-Hopfield ones) may be promoted to the sidebands. The most prominent cases are the blue peak appearing at around 1.8 eV (contributing to SB12 *A*^2^Π*_u_*) and the red peak at 3.2 eV (contributing to SB12 $X^{2}{\text{Σ}}_{g}^{+}$).

**Fig. 4.**
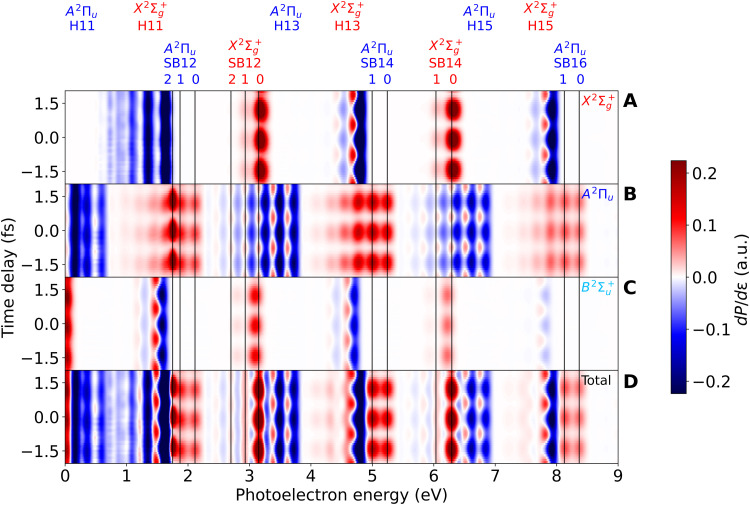
Channel resolved N_2_ RABBIT spectra. (**A**) $X^{2}{\text{Σ}}_{g}^{+}$ channel. (**B**) *A*^2^Π*_u_* channel. (**C**) $B^{2}{\text{Σ}}_{u}^{+}$ channel. (**D**) Total RABBIT spectrum. The vertical thin black lines indicate the position of the vibrational states associated with the different ionization channels. The spectra have been convoluted with a Gaussian of 80 meV FWHM to account for the experimental resolution of (*34*).

The calculated RABBIT spectra obtained by varying the delay between the APT and the IR pulse is shown in Fig. 4, for each individual ionization channels and for the sum over all channels. As in Fig. 3C, the spectra have been referred to those obtained with the APT only, so that one can better identify the contribution of the sidebands. As expected, all sideband peaks oscillate with a frequency that is approximately twice the frequency of the IR pulse, but the relative phases are different for different types of peaks. The vertical lines across the whole figure indicate the expected position within the sidebands of the different vibrational peaks associated with the $X^{2}{\text{Σ}}_{g}^{+}$ and *A*^2^Π*_u_* channels after absorption (or emission) of an IR photon. In particular, the peaks with *v*′ ≥ 3 associated with the SB12$\left(X^{2}{\text{Σ}}_{g}^{+}\right)$ + H13(*A*^2^Π*_u_*) band contain information about the narrow Feshbach resonances lying just below the *A*^2^Π*_u_* threshold, which come from the H11$\left(X^{2}{\text{Σ}}_{g}^{+}\right)$ band after absorption of an IR photon. In addition, the *v*′ = 1 and *v*′ = 2 peaks carry information about the lowest two Hopfield resonances lying just above the *A*^2^Π*_u_* threshold. Because of the rather long lifetime of all these resonances, specially those lying just below the *A*^2^Π*_u_* threshold, absorption of the IR photon mostly occurs before the resonances have effectively decayed by autoionization, so that the dominant effect of the IR pulse is to promote population into the electronic continuum where the SB12$\left(X^{2}{\text{Σ}}_{g}^{+}\right)$ + H13(*A*^2^Π*_u_*) band appears. As a consequence, the relative intensities of the different *v*′ peaks in the SB12$\left(X^{2}{\text{Σ}}_{g}^{+}\right)$ + H13(*A*^2^Π*_u_*) band looks different from that in the SB14$\left(X^{2}{\text{Σ}}_{g}^{+}\right)$ + H15(*A*^2^Π*_u_*) band. This information is also imprinted in the sideband phases. Similar effects reflect on the *v*′ = 0 and *v*′ = 1 peaks associated with the SB12(*A*^2^Π*_u_*) + H11$\left(X^{2}{\text{Σ}}_{g}^{+}\right)$ band, although, in this case, the information is basically restricted to the lowest two Hopfield resonances lying just above the *A*^2^Π*_u_* threshold. All these features are somewhat diluted when one looks at the total RABBIT spectrum, which is what was actually measured in (*34*).

**Fig. 5.**
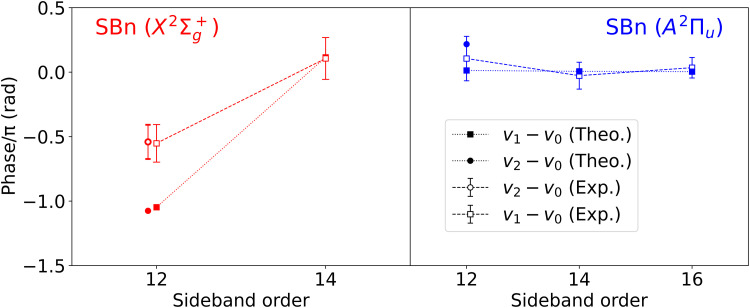
Vibrationally resolved photoionization phases. Full symbols: phases resulting from fitting the sidebands appearing in the total RABBIT spectrum shown in Fig. 4D to a cosine function of frequency 2ω*_IR_* in an energy interval of 10 meV around the expected position of vibrational peaks associated with the $X^{2}{\text{Σ}}_{g}^{+}$ and *A*^2^Π*_u_* channels (vertical lines in Fig. 4). Experimental results of Haessler *et al.* (*34*, *56*) are shown by open symbols with their corresponding error bars. The dashed and dotted lines are plotted to guide the eye. All phases are referred to the phase of the *v* = 0 peak in the corresponding ionization channel and the corresponding SB. The right vertical axis shows the correspondence between calculated phases and photoionization delays.

To compare with the latter experimental work, we have fitted the regions of the total spectrum corresponding to the positions of the vibrational peaks indicated in Fig. 4 to the usual cosine function with frequency 2ω*_IR_* and extracted the relative phases. The results are shown in Fig. 5. The corresponding absolute phases are shown in fig. S2. The bars in the calculated values of the phases indicate the statistical errors resulting from the fit. As can be seen, the agreement between theory and experiment is remarkable. In particular, the abrupt change of the phase when going from SB14$\left(X^{2}{\text{Σ}}_{g}^{+}\right)$ to SB12$\left(X^{2}{\text{Σ}}_{g}^{+}\right)$ is well described by the calculations. This phase change amounts to ∼600 as when converted to units of time (∼300 as in the experiment). The discrepancy in the absolute value for SB12$\left(X^{2}{\text{Σ}}_{g}^{+}\right)$ can be due to the chosen range of photoelectron energies to perform the fit, which may be different from that used in the experiment, or due to small errors in the relative positions of the many spectral features that overlap in this region. None of these have a substantial effect in the phases that vary smoothly with photoelectron energy. In the case of the SB12(*A*^2^Π*_u_*) band, a substantial phase change is only visible for *v*′ = 2 (∼140 as), although, in this case, there are no experimental results to compare with.

**Fig. 6.**
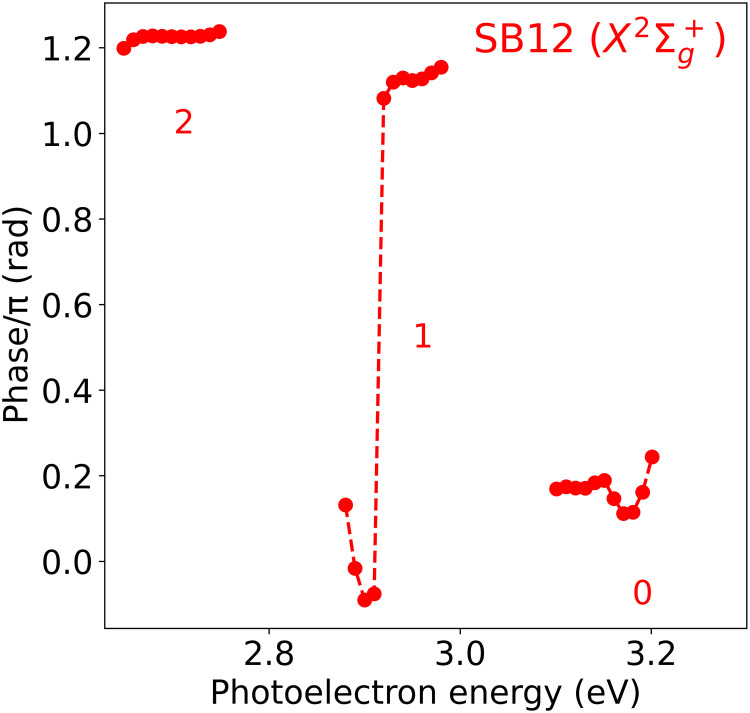
Energy-resolved photoionization phases. Absolute phases resulting from fitting the SB12 $X^{2}{\text{Σ}}_{g}^{+}$ sideband appearing in the total RABBIT spectrum shown in Fig. 4D to a cosine function of frequency 2ω*_IR_* in energy intervals of 10 meV. Numbers 0, 1, and 2 indicate the energy regions associated with the different vibrational states *v* of the remaining N${}_{2}^{+}$ cation.

Since it is nowadays possible to extract phase variations across sidebands due to the improved energy resolution of the experiments (the so-called rainbow RABBIT), Fig. 6 shows the actual variation of the phase within SB12 $X^{2}{\text{Σ}}_{g}^{+}$. As can be seen, the phase varies by about π around 2.9 eV and by about 0.1π around 3.2 eV in narrow energy intervals. These variations are partly due to the signature of the lowest Hopfield resonance in SB12 $X^{2}{\text{Σ}}_{g}^{+}$ [the red peak in fig. S1 (B and C)] as well as to the contribution of the other ionization channels, *A*^2^Π*_u_* and $B^{2}{\text{Σ}}_{u}^{+}$, in the same energy region. Therefore, to extract the phase variation associated with the lowest Hopfield resonance in the $X^{2}{\text{Σ}}_{g}^{+}$ channel, one must design experimental strategies to remove the contribution from the latter channels. As we will see below, this can be achieved by performing angularly resolved measurements. The results shown in Fig. 6 also show that the discrepancy between experiment and theory for SB12 $X^{2}{\text{Σ}}_{g}^{+}$ (see Fig. 5) can be due to the energy averaging of the phase over different spectral ranges. Similar plots for other sidebands can be found in the Supplementary Materials.

It is important to stress here that in energy regions where the overlap of harmonic bands and sidebands cannot be completely removed, or several ionization channels and many resonances contribute, one cannot always relate the measured phase variations to actual photoionization delays. As shown above, it is clearly the case for SB12 $X^{2}{\text{Σ}}_{g}^{+}$ at around 2.9 and 3.2 eV (associated with apparent final vibrational states *v* = 1 and *v* = 0 of the $X^{2}{\text{Σ}}_{g}^{+}$ state, respectively).

**Fig. 7.**
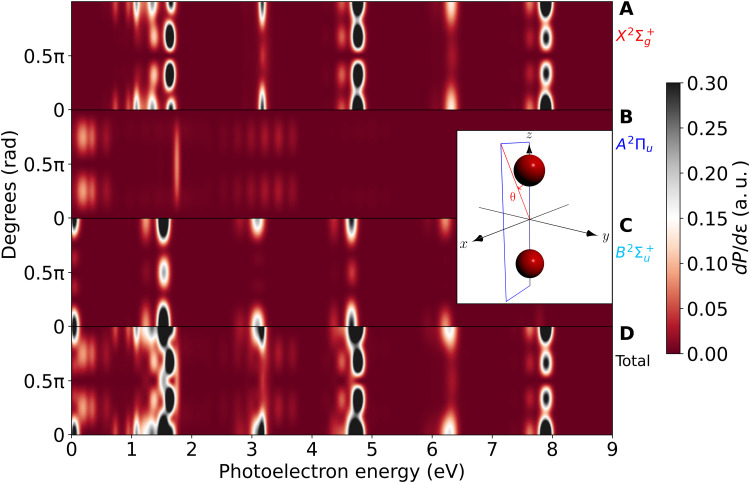
Angularly resolved spectra for molecules oriented along the polarization direction. Photoelectron angular distributions for N_2_ molecules parallel to the polarization direction (*z* axis) for no delay between the APT and the IR pulse. The angular distributions are shown in the plane that contains the molecular axis (*z*) and the *x* axis, and the observation angle is θ, as defined in the inset. (**A**) $X^{2}{\text{Σ}}_{g}^{+}$ channel. (**B**) *A*^2^Π*_u_* channel. (**C**) $B^{2}{\text{Σ}}_{u}^{+}$ channel. (**D**) Total. The spectra have been convoluted with a Gaussian of 80-meV FWHM to account for the experimental resolution of (*34*).

All previous results show that the complexity of RABBIT spectra in the vicinity of molecular Feshbach resonances can be accurately described by correctly accounting for electron correlation, nuclear motion, and the coupling between nuclear and electronic degrees of freedom, beyond the Born-Oppenheimer approximation. However, this complexity, which reflects in the many overlapping spectral features, may prevent one from extracting photoionization delays from real experiments. To solve this problem, current experimental efforts are pursuing to combine the RABBIT technique with alignment (*37*) or multicoincidence detection techniques (*22*, *23*, *25*, *35*) that can provide photoelectron spectra for a given molecular orientation, thus reducing the number of accessible channels. In addition, if one has access to the photoelectron angular distributions, which is now the state of the art in atomic RABBIT, the number of accessible channels can be further decreased, thus allowing for a more clear isolation of the spectral features of interest. Having this in mind, we have calculated photoelectron spectra resolved in both electron emission angle and molecular orientation. As an illustration, Fig. 7 shows the photoelectron angular distributions for N_2_ molecules parallel to the polarization direction (*z* axis) for no delay between the APT and the IR pulse. The upper three panels show the results for the individual ionization channels, and the bottom panel shows their sum. As can be seen, the lines associated with the Feshbach resonances contributing to the SB12$\left(X^{2}{\text{Σ}}_{g}^{+}\right)$ band at around 3.2 eV are perfectly isolated from any other feature in the $X^{2}{\text{Σ}}_{g}^{+}$ channel and reaches its maximum intensity at an electron emission angle of 90^∘^. This feature is nearly invisible in the integrated spectrum shown in Figs. 3B and 4A, but here, it is quite apparent, even without resolving the final state of the cation, since the vibrational progression associated with the SB12(*A*^2^Π*_u_*) band, which is dominant in the integrated spectrum, is nearly invisible at 90^∘^ (see second topmost panel in Fig. 7). Also, the signature of the lowest Hopfield resonance contributing to the SB12(*A*^2^Π*_u_*) band at around 1.8 eV can be clearly seen, both in the spectra associated with the *A*^2^Π*_u_* channel and at 90^∘^ in the spectra in which the individual channels are not resolved.

In conclusion, we have implemented a theoretical tool to describe ionization of many-electron molecules irradiated by a combination of XUV and IR ultrashort pulses in the vicinity of Feshbach resonances. A correct description of this process requires accounting for electron correlation in the electronic continuum, the coupling between nuclear and electronic motions, and the evaluation of bound-bound, bound-continuum, and continuum-continuum transition matrix elements between fully correlated states, all of the above in the time domain. The methodology has been used to calculate time-resolved and vibrationally resolved photoelectron spectra of N_2_ by varying the delay between an APT and an IR pulse following the premises of the RABBIT technique. Our results, in very good agreement with those of an early and unique experiment reported in 2009 (*34*), show that Feshbach resonances are responsible for unusual non–Franck-Condon vibrational progressions and abrupt phase variations in the sideband phases. The presence of a forest of long-lived Feshbach resonances lying just below one of the ionization thresholds leads to an increase of the peak intensities in the energy regions where the remaining molecular cation is in higher vibrational states. It also leads to an apparent jump in the photoionization delays that can be as large as 600 as. This should not be interpreted, however, as a true photoionization delay, as it results from the superposition of different autoionization channels in the low-energy region of the spectrum, but as a clue of the effective population of Feshbach resonances by the incoming light. Hence, theoretical input on at least the potential energy curves and autoionization lifetimes of potentially accessible Feshbach resonances is almost mandatory to achieve a complete understanding of the experimental measurements. Such information can be provided by accurate electronic structure methods such as XCHEM (*47*), which is at the heart of the methodology presented in this work. Our results also show that, to isolate some of the multiple overlapping features appearing in the spectra and obtain temporal information from them, one would rather perform angularly resolved RABBIT measurements for a fixed molecular orientation. Current experimental efforts are pursuing this goal by combining RABBIT setups with multicoincidence detection methods or by aligning the molecule with an auxiliary laser [see, e.g., (*22*, *23*, *25*, *35*, *37*)].

## METHODS

We have solved the time-dependent Schrödinger equation (atomic units will be used from now on)

$$i\frac{\partial \text{Ψ}(\vec{r},R,t)}{\partial t}=H\left(t\right)\text{Ψ}(\vec{r},R,t)$$ (1)

where $\vec{r}$ and *R* refer to the electronic and nuclear coordinates, respectively, *t* represents the time, Ψ($\vec{r}$, *R*, *t*) is the time-dependent wave function, and ℋ(*t*) is the time-dependent Hamiltonian given by

$$H(t)={\hat{H}}_{0}+\hat{T}+\vec{E}(t)\cdot \vec{d}+V_{CAP}$$ (2)

where ${\hat{H}}_{0}$ is the field free electronic Hamiltonian, $\hat{T}$ is the nuclear kinetic energy operator, $\vec{E}(t)\cdot \vec{d}$ is the laser-molecule interaction potential in length gauge with $\vec{E}(t)$ being the electric field and $\vec{d}=\sum_{i}{\vec{r}}_{i}$, and *V_CAP_* is a complex absorbing potential that avoids nonphysical electronic reflections at the box boundary. The Ψ($\vec{r}$, *R*, *t*) wave function is written as an expansion over products of diabatic electronic states $\psi_{i}^{D}(\vec{r},R)$, which depend parametrically on the internuclear distance, and nuclear wave functions χ*_i_*(*R*, *t*) that carry the time dependence:

$$\text{Ψ}(\vec{r},R,t)=\sum_{i}\psi_{i}^{D}(\vec{r},R)\chi_{i}(R,t)$$ (3)

 

The use of a basis of diabatic electronic states instead of adiabatic ones is numerically convenient to guarantee that dipole transition matrix elements vary smoothly with *R* and that nonadiabatic effects are taken into account without including nonadiabatic couplings that may vary very rapidly with *R*. The price to pay is that one has to include Hamiltonian couplings, since diabatic electronic states are not eigenfunctions of the electronic Hamiltonian, but these are, in general, easy to calculate and do not change abruptly with *R*. In our calculations, we have neglected molecular rotation, which is justified because they are more than three orders of magnitude slower than the investigated electron dynamics. Thus, in the center of mass of the molecule, the set of nuclear coordinates *R* reduces to one dimension: the internuclear distance.

The TDSE is solved by using the split-operator method (*48*–*50*), in combination with fast-Fourier techniques (*51*). In brief, the evolution operator is written as

$$U(t+\text{Δ}t)=e^{-\frac{iT\text{Δ}t}{2}}e^{-\frac{i\vec{E}(t)\cdot \vec{d}\text{Δ}t}{2}}e^{-i(H_{0}+V_{CAP})\text{Δ}t}e^{-\frac{i\vec{E}(t)\cdot \vec{d}\text{Δ}t}{2}}e^{-i\hat{T}\text{Δ}t2}$$ (4)

where we use a time step Δ*t* = 10 as. The nuclear wave packets are discretized on a grid of equidistant *R_k_* points separated by Δ*R*

$$\chi_{i}(R,t)=\sum_{k}^{N}\delta (R-R_{k})\chi_{i}(R_{k},t)\text{Δ}R$$ (5)

and their equivalent forms in momentum space to evaluate the kinetic energy terms. Propagation of the wave packets is performed on a set of coupled electronic states calculated on the same grid.

The ionization probability for a given electron energy ϵ and a given final vibrational state of the molecular cation can be obtained by projecting Ψ($\vec{r}$, *R*, *t*) onto a product of an electronic continuum state fulfilling the exact multichannel incoming boundary conditions ${\text{Ψ}}_{\alpha \epsilon \ell \sigma }^{-}(\vec{r},R)$(i.e., a scattering state) and the vibrational wave function of the remaining cation ϕ_ν_α__(*R*). Here, α represents the electronic state of the remaining molecular cation, 𝓁 denotes a given partial wave, and σ indicates the spin of the ejected electron. As we are interested in obtaining photoelectron spectra irrespective of the final vibrational state of the remaining cation, which is what is measured in most RABBIT experiments, as, e.g., in (*34*), one has to sum over all possible final vibrational states, including dissociative states

$$P_{\alpha \epsilon \ell \sigma }=\sum_{\nu }|\int dR\int dr\;{\text{Ψ}}_{\alpha \epsilon \ell \sigma }^{-*}(\vec{r},R)\phi_{\nu_{\alpha }}(R)\text{Ψ}(\vec{r},R,t_{f}){|}^{2}$$ (6)

where *t_f_* is the integration time. After some trivial algebra, this ionization probability is given by

$$P_{\alpha \epsilon \ell \sigma }=\sum_{k}\text{Δ}R|A(R_{k},t_{f}){|}^{2}$$ (7)

where Δ*R* is the difference between two consecutive grid points, and

$$A(R_{k},t_{f})=\sum_{i}\chi_{i}(R_{k},t_{f})\int dr{\text{Ψ}}_{\alpha \epsilon \ell \sigma }^{-*}(\vec{r},R_{k})\psi_{i}^{D}(\vec{r},R_{k})$$ (8)

Notice that, in Eq. 7, the explicit form of the ϕ_ν_α__ functions is not needed. The ionization probability in a given electron emission direction in the molecular frame is obtained as described in (*52*).

We performed TDSE calculations for molecules oriented parallel and perpendicular to the polarization direction of the applied fields. Photoelectron spectra for randomly oriented molecules were approximately evaluated by weighting the results for the parallel and the perpendicular orientations with factors 1/3 and 2/3, respectively. In Eq. 3, we have included all electronic states, bound and continuum ones, that can be reached up to a photon energy of 63 eV, which amounts to 21,039 electronic states for the perpendicular orientation and 21,000 for the parallel orientation.

### Electronic states and couplings

Adiabatic electronic states and the corresponding dipole couplings between them were calculated with the XCHEM code (*46*, *47*, *52*), which makes use of a hybrid Gaussian/B-spline basis and a close-coupling approach to describe the ionization continuum. In this work, we have included the $X^{2}{\text{Σ}}_{g}^{+}$, *A*^2^Π*_u_*, and $B^{2}{\text{Σ}}_{u}^{+}$ ionization channels. We used a set of polycentric orbitals obtained from state-average restricted active space self-consistent field (SA-RASSCF) calculations with a cc-pVQZ basis (*53*) by using MOLPRO (*54*), in which the 1σ*_g_* and 1σ*_u_* orbitals are doubly occupied; the 2σ*_g_*, 2σ*_u_*, 3σ*_g_*, 3σ*_u_*, 1π*_g_*, and 1π*_u_* orbitals can have an arbitrary number of electrons; and the 4σ*_g_*, 4σ*_u_*, 5σ*_g_*, 5σ*_u_*, 6σ*_g_*, 6σ*_u_*, 2π*_g_*, 2π*_u_*, 3π*_g_*, 3π*_u_*, 1δ*_g_*, and 1δ*_u_* orbitals can have a maximum of two electrons. These orbitals were optimized with respect to the *X*^1^Σ*_g_*, *A*^1^Π*_u_*, *B*^1^Σ*_u_*, and *C*^1^Σ*_u_* states of the neutral molecule and transformed to the MOLCAS format (*55*). In addition, a basis of monocentric basis functions was placed at the center of mass of the molecule. This basis consists of a set of even-tempered Gaussian functions $G_{i}^{M}(r)\propto r^{2k+l}e^{-\alpha_{i}r^{2}}$, where α*_i_* = α_0_β*^i^* (α_0_ = 0.01,  β = 1.46,  *i* = 0,1, …,21), with *l* ≤ 3 and *k* ≤ 2, and a set of 3488 B-splines of order 7 located in between *r*_min_ = 7 a.u. and *r*_max_ = 1800 a.u. The CAP, defined as −*i*10^−4^(*r* − 1750)^2^, was placed at 1750 a.u.

### Diabatization

We transformed the set of adiabatic states obtained with XCHEM into a set of diabatic states belonging to different subspaces, each one defined by a parent ion state, and added an additional subspace containing all adiabatic bound states. In this diabatic representation, the Hamiltonian is diagonal in each subspace, and all states have a well-defined character. All Hamiltonian couplings between diabatic states belonging to different subspaces, as well as dipole couplings between them, appear as off-diagonal terms. In this way, both Hamiltonian and dipole couplings vary smoothly with *R*, because (i) the many avoided crossings between the potential energy curves (PECs) of the resonances appearing at small R are transformed into real crossings, and (ii) the interchannel couplings between the different states of the molecular cation is turned off. The effect of these couplings is thus introduced through the solution of the TDSE. In the Franck-Condon region, where most of the ionization takes place, the variation of these couplings with *R* is small. Thus, to reduce the computational effort without compromising the accuracy, we assumed that these couplings are constant. Of course, the variation with *R* of the diagonal terms of the Hamiltonian, i.e., the PECs, was kept. In more detail, off-diagonal terms were obtained with XCHEM at the equilibrium geometry (1.1 Å), and the PECs were evaluated in a grid of 30 internuclear distances in the interval [0.85 to 1.38] Å by using MOLPRO (*54*) at the multireference configuration interaction (MRCI) level after a complete active space self-consistent field (CASSCF) calculation in which we considered the 1σ*_g_* and 1σ*_u_* orbitals doubly occupied and the 2σ*_g_*, 2σ*_u_*, 3σ*_g_*, 3σ*_u_*, 1π*_g_*, and 1π*_u_* orbitals with an arbitrary number of electrons.
