# Supplementary material for: Attosecond photoionization delays in the vicinity of molecular Feshbach resonances
Source: Sci Adv. 2023 Apr 12;9(15):eade3855. doi: 10.1126/sciadv.ade3855 (PMC10096576; doi:10.1126/sciadv.ade3855)
Supplement: Supplementary file 1 — Figs. S1 to S3 [file sciadv.ade3855_sm.pdf]

Science Advances
AAAS

# Supplementary Materials for

## Attosecond photoionization delays in the vicinity of molecular Feshbach resonances

Vicent J. Borràs *et al.*

Corresponding author: Fernando Martín, fernando.martin@uam.es

*Sci. Adv.* **9**, eade3855 (2023)
DOI: 10.1126/sciadv.ade3855

**This PDF file includes:**

Figs. S1 to S3

# Unconvoluted spectra resulting after 50 fs time integration

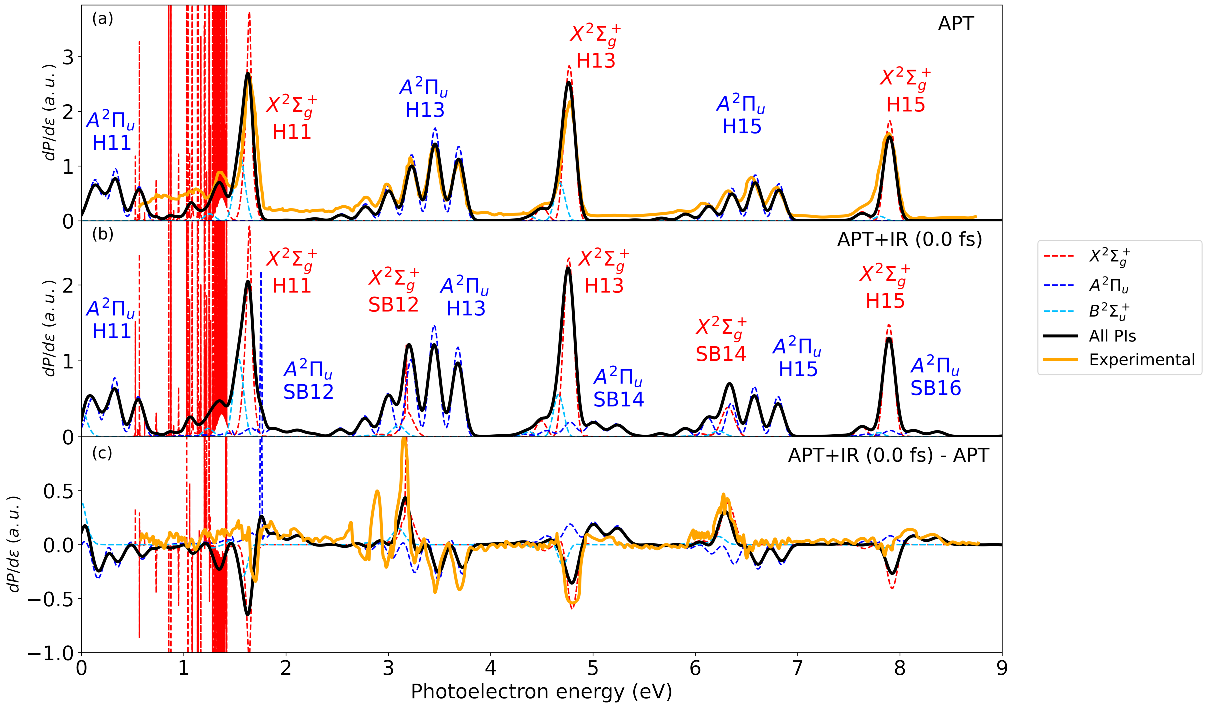


FIG. S1: **Photoelectron spectrum plus population remaining in the Feshbach resonances after 50 fs time integration.** Contribution of the different ionization channels and Feshbach resonances to the (a) APT-only and (b) APT+IR spectra at zero delay between the APT and the IR , and (c) the corresponding difference, when the TDSE is integrated up to 50 fs and no convolution is performed. In this short time interval, the accessible resonances converging to the $A^2\Pi_u$ threshold (blue curves in Fig. 1) appear as narrow spikes because they have not had enough time to decay by autoionization, while those lying just above the $A^2\Pi_u$ threshold (Hopfield resonances, pale blue curves in Fig. 1) have completely decayed.

# Absolute phases extracted from sidebands

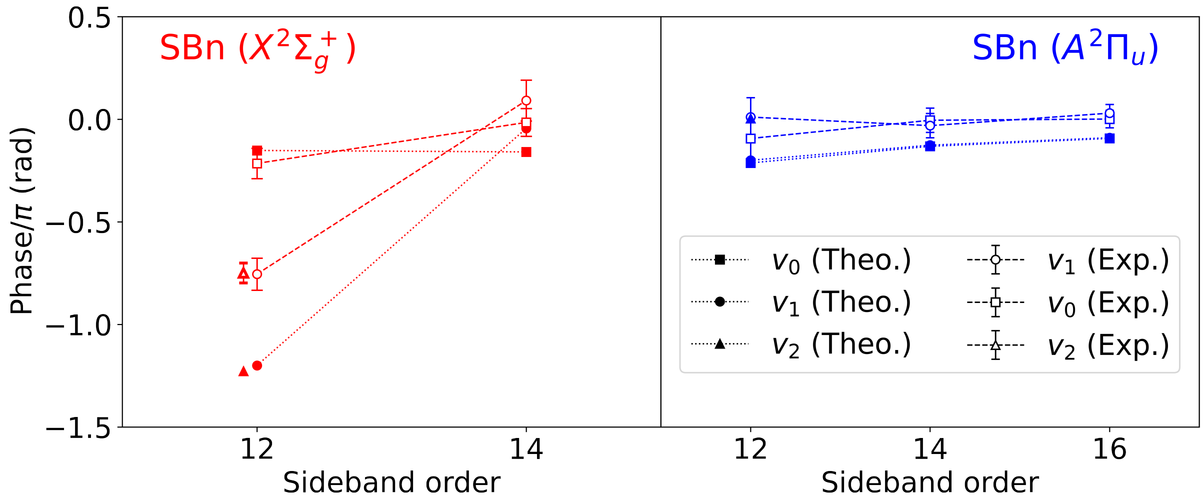


FIG. S2: **Absolute phases.** Absolute phases resulting from fitting the sidebands appearing in the total RABBIT spectrum shown in Fig. 4d to a cosine function of frequency $2\omega_{IR}$ in energy intervals of 10 meV around the different vibrational peaks associated with the $X^2\Sigma_g^+$ and $A^2\Pi_u$ channels (full symbols). Experimental results of Haessler et al *(34,48)* are shown by open symbols with their corresponding error bars. The dashed and dotted lines are plotted to guide the eye. The right vertical axis shows the correspondence between calculated phases and photoionization delays.

Note: The phases originally reported in Ref. *(34)* have been revisited by the authors in a recent erratum *(48)*. The figure shows the results reported in the latter publication.

## Energy resolved phases extracted from sidebands

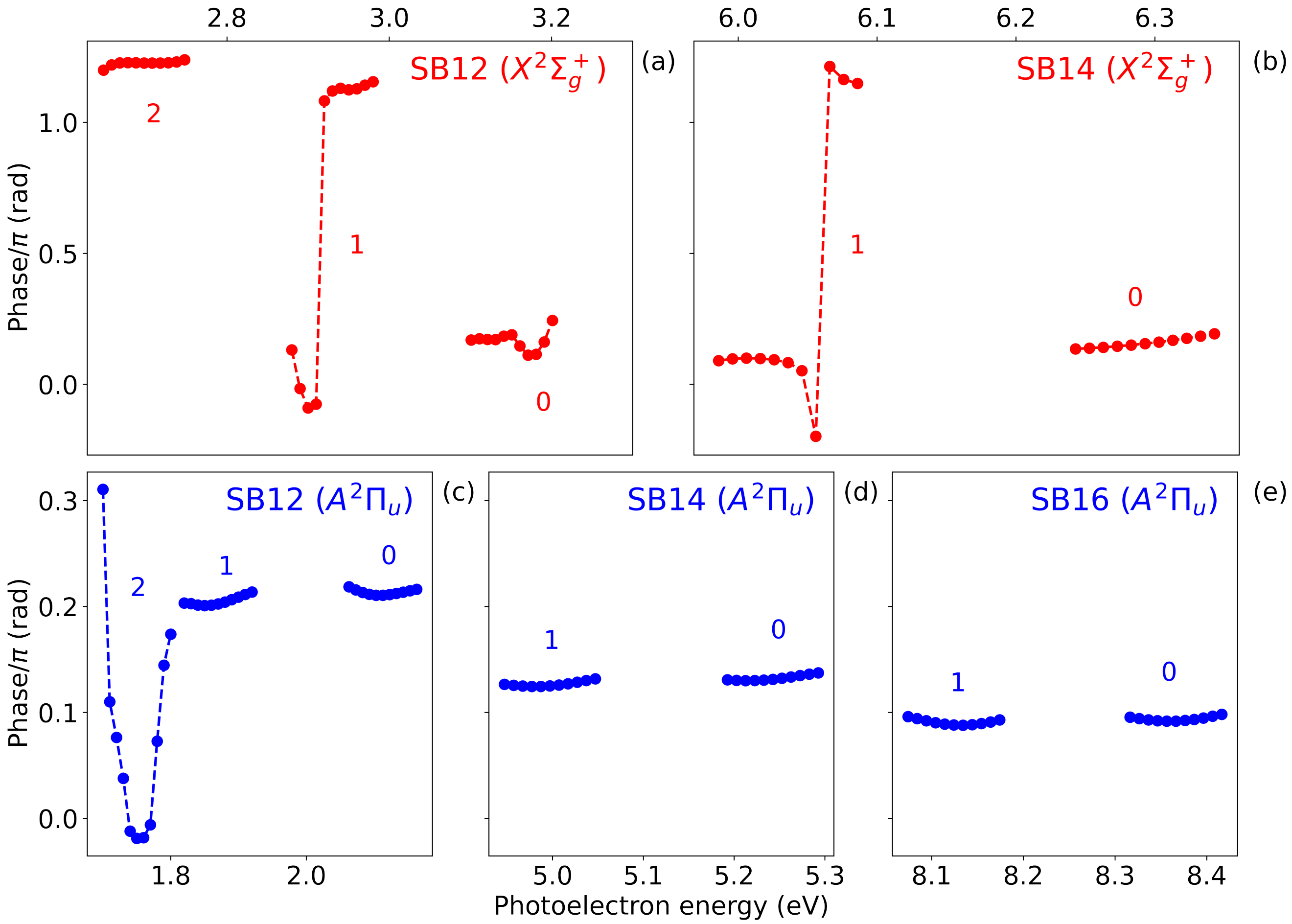


FIG. S3: **Energy resolved phases.** Energy resolved absolute phases resulting from fitting the sidebands appearing in the total RABBIT spectrum shown in Fig. 4d to a cosine function of frequency $2\omega_{IR}$ in energy intervals of 10 meV. Numbers 0, 1 and 2 indicate energy regions associated with different vibrational states $v$ of the remaining $N_2^+$ cation.
